# Early disease control with a biochemical response to aumolertinib after postoperative recurrence of EGFR-Mutant stage IIIA lung adenocarcinoma after icotinib, with EGFR T790M detected at recurrence: a Case Report

**DOI:** 10.3389/fphar.2026.1878253

**Published:** 2026-10-01

**Authors:** Zhenyu Niu, Yin Zhu, Yu Zhou, Yunhong Xu, Lili Jin, Qibin Shen

**Affiliations:** 1 Huzhou Central Hospital, The Affiliated Central Hospital of Huzhou Normal University, Huzhou, Zhejiang, China; 2 Huzhou Central Hospital, The Fifth School of Clinical Medicine of Zhejiang Chinese Medical University, Huzhou, Zhejiang, China; 3 Department of Central Laboratory, Huzhou Central Hospital, Affiliated Central Hospital of Huzhou Normal University, Huzhou, Zhejiang, China; 4 Department of Thoracic Surgery, Huzhou Central Hospital, Affiliated Central Hospital of Huzhou Normal University, Huzhou, Zhejiang, China

**Keywords:** acquired resistance, aumolertinib, EGFR T790M, epidermal growth factor receptor-tyrosine kinase inhibitors (EGFR-TKIs), non-small cell lung cancer (NSCLC)

## Abstract

**Background:**

Epidermal growth factor receptor tyrosine kinase inhibitors (EGFR-TKIs) are a standard treatment for EGFR-mutant non-small cell lung cancer (NSCLC). However, acquired resistance commonly develops after treatment with first- or second-generation EGFR-TKIs. Aumolertinib, a third-generation EGFR-TKI, has demonstrated efficacy in tumors harboring the EGFR exon 20 T790M resistance mutation.

**Case Presentation:**

A 57-year-old man with stage IIIA lung adenocarcinoma and a history of hypertension and long-term smoking underwent thoracoscopic lobectomy with intrapulmonary lymph node dissection. Postoperative molecular testing identified an EGFR exon 19 deletion, and adjuvant oral icotinib (375 mg/d) was initiated. After treatment discontinuation, disease recurrence was detected on PET-CT involving the right hilar lymph nodes and a right pleural/subpleural lesion, which was confirmed by biopsy. Repeat molecular testing revealed concurrent EGFR exon 19 deletion and EGFR exon 20 T790M mutation. After 1 month of aumolertinib treatment, the target lesion decreased in size from 24 to 23 mm and was assessed as stable disease according to RECIST version 1.1, whereas the serum CEA level decreased from 24.19 to 4.35 ng/mL, indicating early disease control with a biochemical response.

**Conclusion:**

This case demonstrates early disease control and a biochemical response to aumolertinib treatment in recurrent NSCLC with an EGFR exon 20 T790M mutation detected at recurrence. It also highlights the importance of repeat molecular testing to guide therapeutic decisions.

## 1 Introduction

Lung cancer remains a major public health issue and is the leading cause of cancer-related mortality worldwide, with the highest mortality rate among all cancers (Siegel et al., 2023). Owing to the lack of early symptoms, many patients are often diagnosed at advanced or metastatic stages, when treatment options are limited. Non-small cell lung cancer (NSCLC) accounts for 80%–85% of all lung cancer cases. Traditional chemotherapy has limited efficacy, with a 5-year survival rate of 20% (Sung et al., 2021). Clinical treatment options for NSCLC mainly include surgery, chemotherapy, radiotherapy, immunotherapy, and targeted therapy (Reck et al., 2021). With advances in genetic testing, targeted therapy has gained increasing attention. Alterations in the epidermal growth factor receptor (EGFR), a transmembrane receptor tyrosine kinase protein involved in tumor cell proliferation, angiogenesis, tumor progression, metastasis, and cell survival, are common in NSCLC. These mutations are identified in almost 50% of Asian patients (Popat et al., 2022) and are susceptible to treatment with small-molecule EGFR tyrosine kinase inhibitors (TKIs) (Riely et al., 2024). Approximately 80%–90% of these mutations consist of exon 19 deletions (Ex19del) and L858R substitution in exon 21, which are collectively referred to as sensitizing EGFR mutations (Kosaka et al., 2004). Currently, EGFR-TKI molecules are the standard first-line treatment for EGFR mutation-positive NSCLC (Riely et al., 2024). However, most patients develop acquired resistance to first-generation (icotinib, erlotinib, or gefitinib) or second-generation (afatinib/dacomitinib) EGFR-TKIs and experience disease progression within 9–14 months of treatment initiation. Approximately 50% of these cases harbor the T790M mutations (Westover et al., 2018). These T790M resistance mutations are specifically targeted by the third-generation EGFR-TKIs, which irreversibly bind to EGFR and prolong tumor control in patients with NSCLC (Riely et al., 2024).

Aumolertinib (formerly almonertinib; HS-10296), a new third-generation EGFR-TKI, has demonstrated potent anti-cancer activity in phase 1/2 trials involving patients with advanced and metastatic NSCLC carrying sensitive EGFR or acquired T790M mutations (Lu et al., 2022; Yang et al., 2020). It was approved by China’s National Medical Products Administration on 18 March 2020, for the treatment of previously treated patients with NSCLC harboring positive EGFR T790M mutations (Lu et al., 2018).

Here, we report a case of postoperative tumor recurrence in a patient with resected stage IIIA advanced NSCLC harboring an EGFR Ex19Del, in whom an EGFR T790M mutation was detected at recurrence after prior icotinib exposure, and achieved early disease control with a biochemical response after aumolertinib therapy. This case highlights the importance of repeat molecular testing to guide treatment decisions after EGFR-TKI resistance.

## 2 Case presentation

A 57-year-old Chinese man with a history of hypertension and long-term smoking (∼15 cigarettes daily for > 30 years) was admitted on 4 May 2021, after a lung mass was incidentally detected in the right lower lung lobe during a routine physical examination. Chest computed tomography (CT) scans revealed a soft-tissue density mass in the anterior basal segment of the right lower lobe, with suspected obstructive inflammation, as well as a small nodule (∼4 mm in diameter) in the right upper lobe (Figure 1A). Gallstones were also observed. The preoperative serum levels of carcinoembryonic antigen (CEA) were elevated to 36.17 ng/mL.

**FIGURE 1:**
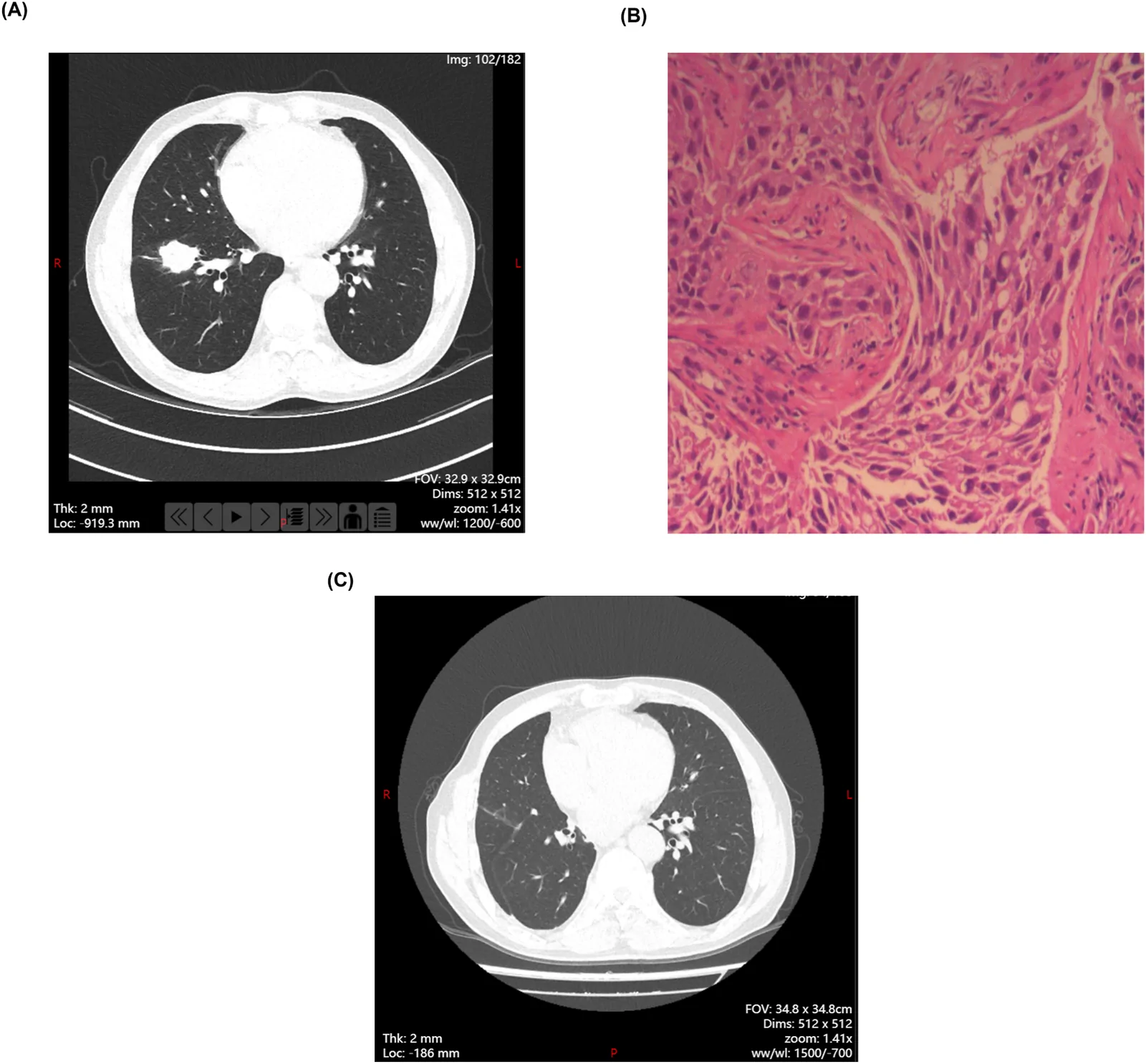
Baseline Disease and Primary Pathology. (**(A)**, Left): Preoperative Contrast-Enhanced Chest CT Scan. Chest CT scan showing a soft-tissue density mass in the anterior basal segment of the right lower lobe at initial presentation. **(B)** Postoperative Pathological Findings. Histopathological examination of the resected right lower lobe tumor showing lung adenocarcinoma and lymph node metastases (hematoxylin and eosin staining). **(C)** Postoperative Follow-Up Chest CT During Adjuvant Icotinib Therapy. Chest CT obtained 5 months after surgery demonstrating no evidence of tumor recurrence.

On 6 May 2021, the patient underwent thoracoscopic right lower lobectomy with mediastinal lymph node dissection. Histopathological examination revealed a peripheral solid adenocarcinoma measuring up to 2.5 × 2.3 × 1.8 cm^3^. The tumor exhibited spread through air spaces (STAS), pleural involvement without penetration, and vascular tumor thrombosis. Lymph node metastases were detected in groups 7 (1 of 4 nodes) and 11 (1 of 1 node), whereas the bronchial margins were negative (Figure 1B). Immunohistochemistry (IHC) showed that tumor cells were negative for squamous cell carcinoma-associated markers cytokeratin (CK) 5/6 and P40 and positive for CK7, thyroid transcription factor 1 (TTF1), and EGFR, suggesting lung adenocarcinoma. Napsin A expression was weakly positive, and the Ki-67 proliferation index was ∼15%. According to the 8th edition of the American Joint Committee on Cancer and the Union for International Cancer Control (AJCC/UICC) TNM staging criteria, the final pathological stage was pT1cN2M0 (stage IIIA). Spread through air spaces (STAS), pleural involvement without penetration, and vascular tumor thrombosis were considered high-risk pathological features for recurrence, but they did not alter the TNM stage.

Postoperative molecular testing was performed using DNA extracted from formalin-fixed paraffin-embedded (FFPE) primary lung tumor tissue, followed by analysis conducted using the AmoyDx Human EGFR Gene Mutations Fluorescence PCR Diagnostic kit (Amoy Diagnostics, Xiamen, China), based on the amplification refractory mutation system combined with dual-ring probe real-time PCR technology (ARMS-PCR), which identified an EGFR Ex19del mutation.

Adjuvant platinum-based chemotherapy was discussed but declined by the patient because of concerns regarding toxicity. Icotinib was selected through shared decision-making, based on the presence of an EGFR Ex19del, drug accessibility, local clinical practice, and patient preference. A planned 3-year treatment course was adopted as an individualized treatment strategy. The patient initiated oral icotinib therapy (375 mg/d) on 20 June 2021. After completing the planned 3-year adjuvant treatment course, icotinib was discontinued in July 2024. Follow-up chest CT performed 5 months after surgery showed only postoperative changes and minimal pleural effusion (Figure 1C), and the serum CEA levels had normalized to 4.19 ng/mL. Subsequent routine follow-up assessments, including chest CT scans and tumor marker evaluations, demonstrated no evidence of recurrence. In November 2025, ∼16 months after the discontinuation of icotinib, disease recurrence was identified on a whole-body positron emission tomography-CT (PET-CT) scan. The scan revealed increased fluorodeoxyglucose (FDG) uptake in the right hilar lymph nodes (SUVmax 6.13) and a right pleural/subpleural nodule measuring ∼12 × 17 mm in the right lower thoracic region, with increased FDG metabolism, considered metastatic recurrence (Figure 2A). This lesion was subsequently evaluated by contrast-enhanced chest CT before the initiation of aumolertinib. The prostate showed benign prostatic hyperplasia with mild FDG uptake (SUVmax, 3.50). Biopsy of the subpleural mass confirmed adenocarcinoma (Figure 2B). IHC revealed negativity for CK5/6, P40, Napsin A, and anaplastic lymphoma kinase (ALK, clone D5F3), and positivity for CK7 and TTF-1, indicating pulmonary adenocarcinoma. The Ki-67 proliferation index was ∼20%, and the programmed death-ligand 1 (PD-L1) IHC assay was <1%. Subsequently, the serum CEA level increased to 24.19 ng/mL, and follow-up contrast-enhanced chest CT scan showed the persistence of the same right pleural/subpleural nodule (Figure 3A).

**FIGURE 2:**
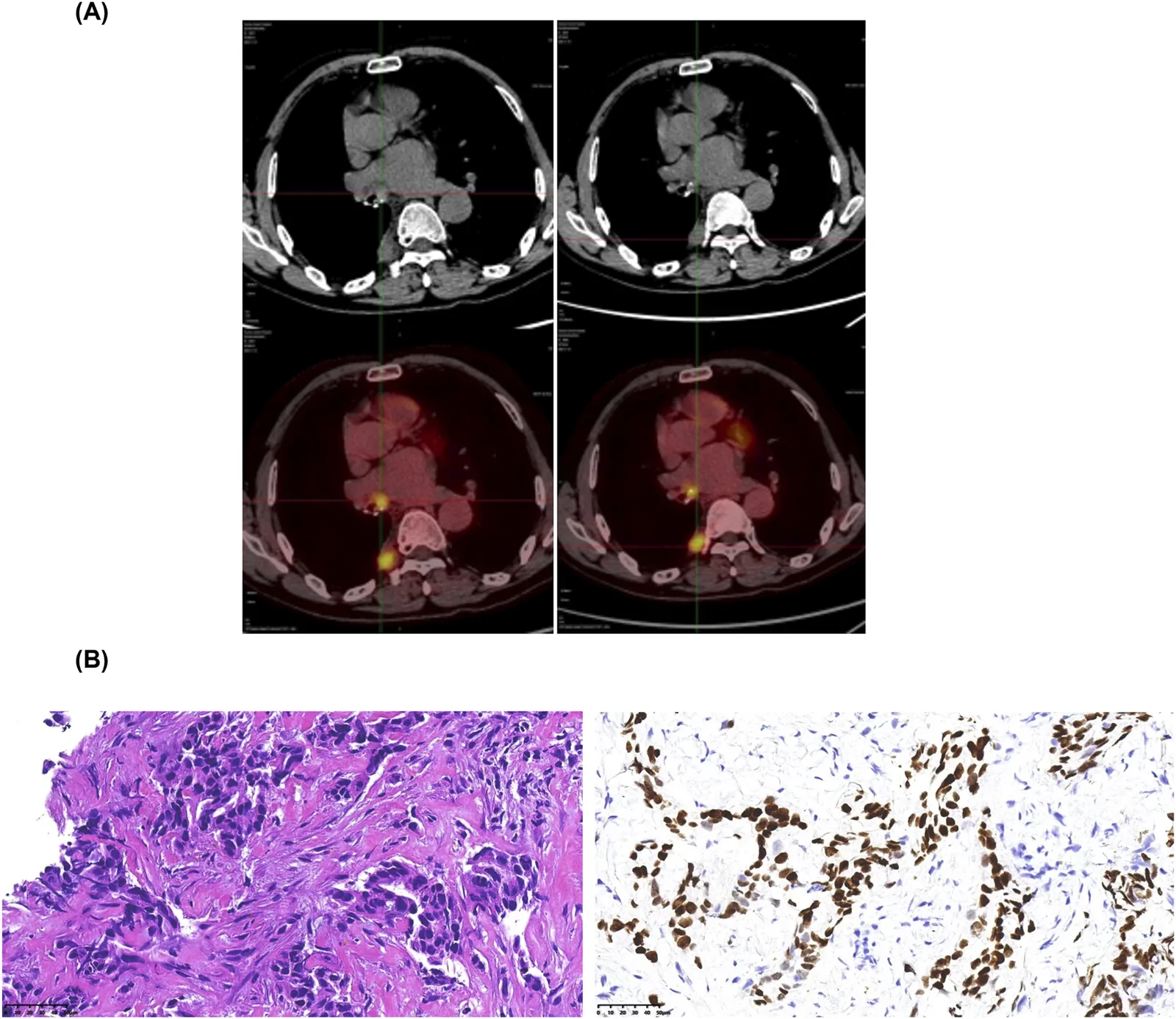
**(A)** PET-CT Findings at Recurrence. Whole-body PET-CT showing increased FDG uptake in the right hilar lymph nodes indicative of metastatic recurrence. **(B)** Pathological Confirmation of Recurrent Disease. Histopathological examination of the biopsy specimen from the right pleural/subpleural lesion confirming recurrent lung adenocarcinoma. (H & E staining, lower left; and IHC assessment, lower right).

**FIGURE 3:**
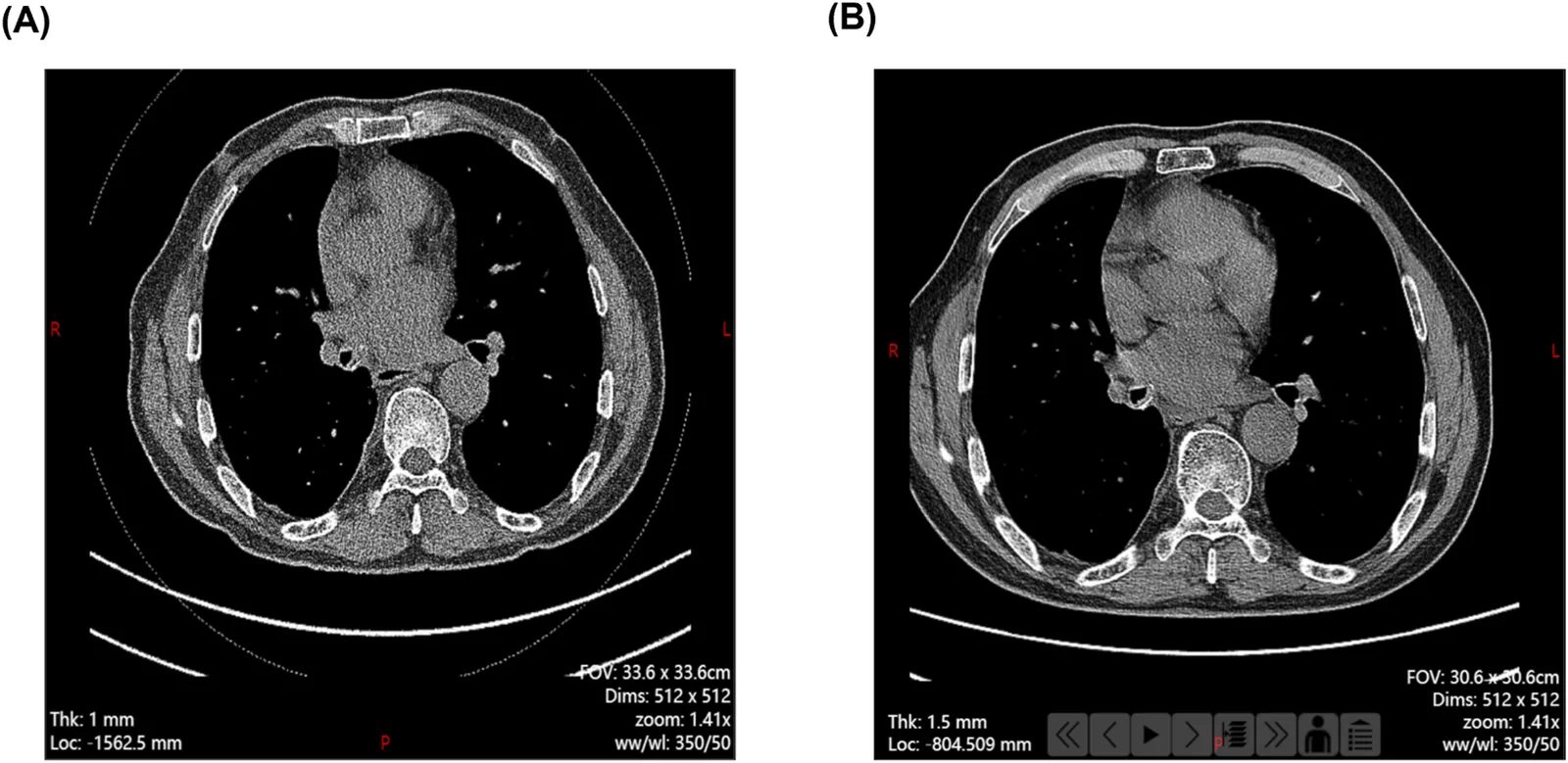
Chest CT before (**(A)**, left) and after (**(B)**, right) aumolertinib treatment.

At recurrence, repeat EGFR mutation testing was performed using DNA extracted from FFPE biopsy tissue of the recurrent subpleural lesion, followed by ARMS-PCR. This testing revealed dual EGFR mutations: Ex19del and exon 20 T790M mutations. The test was conducted according to the manufacturer’s instructions using the clinically validated protocol routinely used in our hospital laboratory as mentioned above. In this study, the ΔCt method was used to indicate the relative abundance of the EGFR mutant allele. Based on these findings, aumolertinib (110 mg/d) was initiated on 22 November 2025. The contrast-enhanced chest CT performed immediately before aumolertinib treatment showed that the longest diameter of the right pleural/subpleural target lesion was 24 mm, which was used as the baseline measurement according to RECIST version 1.1. After 1 month of aumolertinib treatment, the longest diameter decreased to 23 mm. As the reduction (≈4.2%) did not meet the ≥30% threshold for a partial response, the best response was classified as stable disease according to RECIST version 1.1 (Figure 3B).

During adjuvant treatment with icotinib, the patient developed severe skin rash, liver function abnormalities, and mild diarrhea. After symptomatic treatment and temporary discontinuation of icotinib, drug-related adverse events gradually improved. After switching to aumolertinib, the patient experienced only mild skin pruritus and liver function abnormalities, with no diarrhea or other severe adverse events. Liver function was closely monitored and remained stable throughout treatment. Overall, aumolertinib treatment was well tolerated. A schematic timeline of the patient’s clinical course is presented in Figure 4.

**FIGURE 4:**
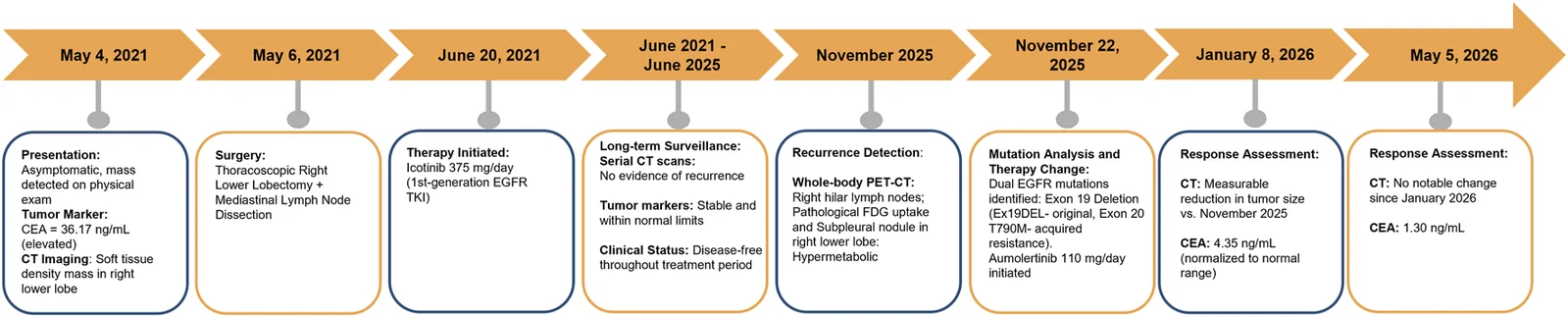
Schematic timeline of the Patient’s clinical course.

## 3 Discussion

The discovery of genes associated with lung cancer and the development of targeted treatments have markedly improved outcomes in patients with driver mutation-positive NSCLC compared with conventional chemotherapy. EGFR-mutant NSCLC represents a biologically distinct subtype, with Ex19del and L858R mutations being the most common sensitizing alterations and strong predictors of response to EGFR-TKIs (Wu et al., 2015; Zhou et al., 2011; Wu et al., 2014; Zhang et al., 2020). Currently, the approved therapies for patients with advanced EGFR-mutant NSCLC include first-generation EGFR-TKIs such as gefitinib, erlotinib, and icotinib; second-generation EGFR-TKIs including afatinib and dacomitinib; and third-generation EGFR-TKIs such as osimertinib and aumolertinib. More recently, combination therapy with amivantamab (an EGFR-MET bispecific antibody) and lazertinib (a third-generation EGFR-TKI) has demonstrated improved PFS compared with osimertinib and has been approved as the first-line therapy in treatment for advanced EGFR-mutated NSCLC (Cho et al., 2024). Patients receiving first-generation and second-generation TKIs typically experience a median PFS between 9 and 14 months (Zhou et al., 2011; Wu et al., 2014; Shi et al., 2017; Mok et al., 2009). Multiple studies have demonstrated that patients with EGFR-mutant tumors derive more benefit from icotinib treatment than those with wild-type EGFR (Dong et al., 2021; Li et al., 2015; Nong et al., 2013; Yao et al., 2015; Wang et al., 2016). Although both mutations are regarded as predictors of response to EGFR-TKIs, patients with tumors harboring the Ex19del mutation experience better clinical outcomes from EGFR-TKI therapy compared to those with the L858R mutation (Chen et al., 2014; Jackman et al., 2006; Patel et al., 2017; Yang et al., 2015). However, drug resistance after treatment remains a significant challenge, with secondary EGFR mutations being the primary cause, among which the EGFR exon 20 T790M mutation is the most prevalent (Seto et al., 2014). Aumolertinib, a third-generation EGFR-TKI, irreversibly binds to the ATP-binding site of EGFR and demonstrates high selectivity for both EGFR-sensitizing mutations and the EGFR exon 20 T790M resistance mutation. It is approved for treating patients with NSCLC who experience disease progression after other EGFR-TKI therapies and test positive for the EGFR exon 20 T790M resistance mutation (Lu et al., 2019). More recently, aumolertinib demonstrated a disease-free survival benefit in the adjuvant setting of the phase 3 ARTS trial, supporting its expanding role beyond the metastatic setting, and has been approved in China as an adjuvant therapy in resected EGFR-mutated NSCLC (Zhang et al., 2026). However, osimertinib remains the globally accepted standard EGFR-TKI for postoperative use. The evidence stems from the ADAURA trial, which demonstrated significantly prolonged disease-free survival with osimertinib (a third-generation EGFR-TKI) compared with placebo in patients with completely resected stage IB-IIIA, EGFR-mutant NSCLC (Tsuboi et al., 2023).

Platinum-based adjuvant chemotherapy has traditionally been recommended for patients with resected stage II-IIIA NSCLC. However, large-scale clinical trials, including the EVIDENCE, ARTS, and ADAURA studies, support EGFR-TKI therapy over adjuvant chemotherapy in patients with resected EGFR-mutant NSCLC (Tsuboi et al., 2023; Zhang et al., 2026; He J. et al., 2021; He Q. et al., 2021). In the present case, postoperative chemotherapy was discussed, but the patient declined after being informed of its potential benefits and toxicities. Adjuvant icotinib was selected because the tumor harbored an EGFR Ex19del and based on drug accessibility, local clinical practice, patient preference, and the available evidence. The 3-year duration of adjuvant icotinib was an individualized treatment strategy established through shared decision-making, and was not based on an established, evidence-based standard duration. At recurrence, repeat molecular testing identified the EGFR T790M mutation, supporting the subsequent use of the third-generation EGFR-TKI, and treatment with aumolertinib was subsequently initiated.

In this context, this case report is clinically relevant as it describes a patient with postoperative recurrence of stage IIIA lung adenocarcinoma after initial treatment with a first-generation EGFR-TKI therapy (icotinib), followed by genetic test identifying concurrent mutations of EGFR Ex19del and EGFR exon 20 T790M, and subsequent treatment with the third-generation EGFR-TKI resulted in early disease control with a biochemical response. In the present case, the initial tumor pathology was severe as it was a solid-type adenocarcinoma STAS, involving lymph node metastases. Thus, it was biologically aggressive, and the findings provided a biological basis for the patient’s subsequent postoperative recurrence. Furthermore, this case highlights the evolution of the resistance EGFR exon 20 T790M mutation at recurrence under treatment pressure. This progression aligns closely with the typical pattern of resistance development observed in targeted therapy for NSCLC. Consequently, the initial response rate to first-generation TKIs can exceed 70% in these patients. However, ∼50%–60% of patients develop resistance after 8–14 months of treatment, often because of secondary mutations or activation of alternative pathways. The EGFR exon 20 T790M mutation is the leading cause of this resistance, as it increases the affinity of EGFR for ATP, reducing the effectiveness of reversible EGFR-TKIs, thereby leading to treatment resistance (Oxnard et al., 2011; Westover et al., 2018). Detection of this mutation at recurrence provided a strong rationale for switching to a third-generation EGFR-TKI that selectively targets both sensitizing EGFR mutations and EGFR exon 20 T790M resistance mutations detected after prior EGFR-TKI exposure. The resistance pattern observed in this case closely matches these epidemiological trends, making it a representative example of a real-world clinical outcome.

This case report provides practical guidance on managing the T790M resistance gene mutation in patients treated with first-generation targeted therapies and emphasizes the clinical significance of independently developed targeted drugs in China. This case adds real-world evidence regarding postoperative recurrence after prolonged adjuvant icotinib therapy in patients with EGFR-mutant NSCLC. Previous studies have primarily evaluated shorter treatment durations. The phase 3 EVIDENCE trial assessed ∼2 years of adjuvant icotinib versus chemotherapy (He J. et al., 2021), and the phase 2 ICOMPARE trial compared 1 year versus 2 years of adjuvant icotinib (Lv et al., 2023). Therefore, the 3-year duration in the present case exceeded the durations most evaluated in clinical trials and should not be interpreted as a standard adjuvant icotinib regimen. The patient received adjuvant icotinib for 3 years and developed recurrence ∼16 months after treatment discontinuation. It also recommends that patients with EGFR Ex19del who develop disease progression after the first-generation TKI therapy should undergo routine secondary genetic testing to identify the mechanisms of acquired resistance. In addition, repeat biopsy is equally necessary in cases of recurrence. In the current case, the biopsy of the recurrent lesion and the genetic testing provided the rationale for switching the treatment to aumolertinib, which targets both sensitizing EGFR mutations as well as acquired resistance. Afterward, the initial 1-month follow-up chest CT report demonstrated only a slight reduction in lesion size, and the serum CEA level returned to the normal range. According to the RECIST version 1.1 criteria, the radiological response was classified as stable disease. These findings suggest early disease control with a favorable biochemical response. If the EGFR exon 20 T790M mutation is detected, third-generation EGFR-TKIs are the recommended treatment choice.

A notable strength of this case is that it illustrates a real-world clinical course rather than simply demonstrating the efficacy of aumolertinib. This describes postoperative recurrence in a patient who had resected stage IIIA/N2 EGFR Ex19del-positive lung adenocarcinoma with multiple high-risk pathological features, including solid adenocarcinoma, STAS, pleural involvement, vascular tumor thrombi, and mediastinal lymph node metastasis. Furthermore, the patient received a prolonged 3-year course of adjuvant icotinib and developed recurrence ∼16 months after treatment discontinuation. Finally, repeat biopsy and molecular testing at recurrence identified the EGFR T790M mutation, which directly guided subsequent treatment with a third-generation EGFR-TKI.

Although it involves a solitary case, this report adds to the real-world evidence on genetic mutation-associated curated treatment strategies for recurrent EGFR-mutant NSCLC. A limitation of this report is that the 3-year duration of adjuvant icotinib was an individualized treatment strategy; therefore, no conclusions regarding the optimal duration of adjuvant icotinib can be drawn from this single case.

## 4 Conclusion

This report describes a case of postoperative recurrence in a patient with resected stage IIIA lung adenocarcinoma who developed an EGFR exon 20 T790M mutation after prior icotinib exposure. Repeat biopsy and molecular testing enabled prompt initiation of aumolertinib, resulting in early disease control with a biochemical response (CEA level decreased from 24.19 to 4.35 ng/mL). Although limited to a particular case, these findings support the potential role of aumolertinib in recurrent EGFR-mutant NSCLC and highlight the importance of repeat molecular testing to guide treatment decisions. This also highlights the need for more patient specific or individualized treatment strategies for patients with EGFR-mutant NSCLC.

## 5 Patient perspective

The patient was asymptomatic at the initial diagnosis, and the pulmonary lesion was incidentally detected during a routine physical examination. During adjuvant icotinib therapy, the patient developed severe skin rash, liver function abnormalities, and mild diarrhea. These adverse events improved after symptomatic treatment and temporary discontinuation of icotinib. Despite these manageable adverse events, the patient maintained an acceptable quality of life and good treatment adherence. The patient remained asymptomatic at the time of disease recurrence and actively cooperated with further treatment after providing informed consent. During aumolertinib treatment, the patient experienced mild skin pruritus and liver function abnormalities without diarrhea or other severe adverse events. Liver function was closely monitored and remained stable throughout treatment. After 1 month of treatment, the lesion had decreased in size and serum CEA levels returned to the normal range. The patient remained psychologically stable and confident in the treatment. After 5 months of follow-up, no notable change in tumor size was observed. The patient complied well with the prescribed treatment and follow-up schedule.

## Data Availability

The original contributions presented in the study are included in the article/supplementary material, further inquiries can be directed to the corresponding authors.
